# Advanced Nanocomposite Hydrogels for Cartilage Tissue Engineering

**DOI:** 10.3390/gels8020138

**Published:** 2022-02-21

**Authors:** Jianghong Huang, Fei Liu, Haijing Su, Jianyi Xiong, Lei Yang, Jiang Xia, Yujie Liang

**Affiliations:** 1 Department of Spine Surgery and Orthopedics, Shenzhen Second People’s Hospital (First Affiliated Hospital of Shenzhen University, Health Science Center), Shenzhen 518035, China; huangjh20@mails.tsinghua.edu.cn (J.H.); jianyixiong@126.com (J.X.); yiyuanbgs@126.com (L.Y.); 2 Shenzhen International Graduate School, Tsinghua University, Shenzhen 518055, China; 3 Department of Biochemistry, Texas A&M University School of Medicine, Bryan, TX 77807, USA; feiliu007@126.com; 4 Technology R&D Department, Shenzhen Lechuang Medical Research Institute Co., Ltd., Shenzhen 518129, China; haijingsu@126.com; 5 Department of Chemistry, the Chinese University of Hong Kong, Shatin, N.T., Hong Kong SAR, China; jiangxia@cuhk.edu.hk; 6 Department of Child and Adolescent Psychiatry, Shenzhen Kangning Hospital, Shenzhen Mental Health Center, Shenzhen 518020, China

**Keywords:** hydrogels, nanocomposite hydrogels, articular cartilage, tissue engineering

## Abstract

Tissue engineering is becoming an effective strategy for repairing cartilage damage. Synthesized nanocomposite hydrogels mimic the structure of natural cartilage extracellular matrices (ECMs), are biocompatible, and exhibit nano–bio effects in response to external stimuli. These inherent characteristics make nanocomposite hydrogels promising scaffold materials for cartilage tissue engineering. This review summarizes the advances made in the field of nanocomposite hydrogels for artificial cartilage. We discuss, in detail, their preparation methods and scope of application. The challenges involved for the application of hydrogel nanocomposites for cartilage repair are also highlighted.

## 1. Introduction

Articular cartilage tissue damage is a common clinical diagnosis [1], typically caused by sports-related injuries, accidental trauma, or inflammation, including osteoarthritis (OA) and rheumatoid arthritis (RA). The main components of cartilage tissue include water, chondrocytes, type II collagen, and proteoglycans. Unlike most other tissues in the human body, cartilage contains only one cell type, chondrocytes. The most important component of cartilage extracellular matrices (ECM) is water, with a content of up to 60–80% [2]. The movement of water molecules within the fibrous network confers stability to cartilage tissue under a range of pressures. As there are no blood vessels or nerves present in cartilage tissue, the self-healing capacity of damaged cartilage tissue is limited. When the damaged area is large (>4 mm) [3], it will not heal itself. At present, the common methods for cartilage tissue repair involve cartilage or chondrocyte transplantation. However, the cartilage prepared by these methods does not mimic the tissue structure of natural cartilage, and its mechanical properties often cannot meet the normal needs of the tissue. In addition, transplantation can induce donor-site lesions.

Scaffold materials provide more possibilities for cartilage defect repair [4]. Cartilage scaffolds require good biocompatibility, suitable porosity, mechanical strength, and scaffolds with structures similar to the type II collagen structure in the cartilage matrix. The scaffolds have high adhesion, which can promote the aggregation and proliferation of chondrocytes in the material and can maintain the synthetic properties of the hyaline cartilage-like matrix. Among them, hydrogel scaffolds with physiological elasticity, a smooth surface, and a high water content can better simulate the ECM microenvironment of natural cartilage and are promising candidates for cartilage regeneration [5,6]. In addition, aerogel is a porous, sponge-like solid material with an extremely large surface area and low density, which has been the focus of developing tissue-engineering scaffolds using nanoscale fabrication in the future [7,8]. In recent years, significant progress has been made in the preparation of hydrogels containing nanocomposites. These hydrogels are easy to prepare, have desirable mechanical properties, and can enhance the stimulation response by the synergistic effect of nanoparticles; as such, they have attracted much attention in the tissue-engineering field [9].

In this review, we discuss the nanomaterials used to make nanocomposite hydrogels. Recent progress and the advantages of nanohydrogels for cartilage regeneration are summarized.

## 2. Nanocomposite Hydrogels

**Figure 1:**
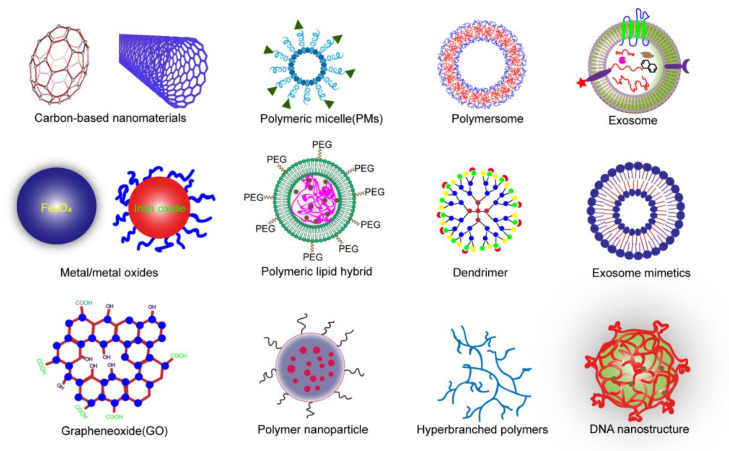
Nanoparticles in NC hydrogels for tissue repair. Carbon-based nanoparticles, metal and metal oxide nanoparticles, polymer nanoparticles, inorganic non-metal nanoparticles, exosomes, exosome analogs, and DNA nanogels that have been reported in recent years.

A nanocomposite (NC) is a form of nanoparticle or carbon nanotubes embedded in a polymer matrix. The nanocomposite hydrogels commonly used for cartilage tissue repair can be classified into the following types: carbon-based material, polymer nanoparticles, metal and metal oxide nanocomposites, inorganic nonmetallic nanoparticles [10], and new modified materials, such as cold-air plasma (Figure 1).

### 2.1. Carbon Nanomaterial Based Hydrogels

Carbon is one of the basic elements of organic life and has been used in many aspects of human society. Since the 20th century, the field of carbon nanomaterials has become more and more popular in tissue engineering. As compared to other tissue-engineering materials, carbon-based nanomaterials have many advantages, such as easy preparation, surface functionalization, good biocompatibility, low cost, and low toxicity. In addition, the strong plasticity of carbon allows carbon-based nanomaterials to be used as secondary structure enhancers to build hybrid materials of metals, ceramics, and polymers to develop stents with optimal performance. Generally speaking, the main types of carbon-based nanomaterials (CBNs) are graphene nanomaterials, carbon nanomaterials, and carbonized polymer nanomaterials. Sui et al. outlined their preparation, properties, and applications in detail [11]

The addition of carbon-based nanomaterial CBNs greatly improves the lubricity of NC hydrogels. For example, Li et al. reported the synthesis of polyethylene glycol (PEG)-grafted CBNs by ultrasonic treatment. The product was measured using a four-ball test at 600 nm without any post-treatment and showed an ultra-low average friction coefficient (0.02) and wear-scar diameter (0.55 mm) [12]. Based on a large number of experimental characterizations and analyses, the proposed mechanisms underlying lubrication in CBNs hydrogels can be divided into four main types: (1) the barrier effect, when CBNs enter the molecular chains of polymer compounds as a spacer to weaken the interactions between molecular chains and cause sliding and rotation and reduce friction; (2) the rolling function, when CBNs act as ball bearings and reduce friction between interfaces; (3) the repair function, when CBNs are deposited onto the wear surface to promote repair minor defects inside the treatment CBNs; (4) the polishing effect, when CBNSs reduce the roughness of surfaces and, thus, reduce friction [13]. The introduction of CBNs into hydrogels can also mimic the lubrication of the synovial tissue in artificial cartilage.

Furthermore, the addition of CBNs has been shown to enhance the mechanical properties of NC hydrogels. For example, Lu et al. prepared injectable composite hydrogels of carbon nanoparticle/polyethylene glycol/chitosan sodium/glycerin phosphate (CBNs/PEG/CS/GP) and found that CBNs, PEG, and GP provided good lubricity through hydrogen bonding and crosslinking [14]. Simultaneously, CBNs enhanced the intermolecular interactions in hydrogels and improved their rheological and mechanical properties. In addition, these materials were easy to prepare and exhibited good biocompatibility. They could, therefore, be considered for use as artificial synovial tissue that could prolong the lifespan of artificial joints.

Graphene oxide nanoparticles (GO-np) have been applied as a new type of inorganic delivery carrier for nanoscale molecular drugs due to their good biocompatibility and adsorption capacity; in addition, they are non-toxic. GO-np has the potential for application in combination with hydrogels. For example, the 3D printing of GO-np hydrogel demonstrated the potential of GO-np in drug delivery, and the hydrogel containing GO-np reduced NF-kB RANK/RANK1 to protect the cartilage tissue [15]. Chen et al. prepared an interpenetrating polymer network of hydrogels with graphene oxide (GO)/polyvinyl alcohol (PVA, inorganic) that was crosslinked with β-cyclodextrin aldehyde (β-CD-DA, organic). These hydrogels exhibited a favorable compression modulus (0.91 MPa), an elongation at break (857%), and biocompatibility [16]. GO was also used to prepare NC hydrogels that were used for cartilage repair. Recently, Cao et al. proposed the use of double-anchoring PEG, in which the hydroxyl group on the PEG chain interacts with the PVA/GO molecules through their hydrogen bonds and their complexes with calcium ions in hyaluronic acid. GO that was not agglomerated and uniformly covered by HA particles was successfully introduced into PVA hydrogels. These results indicated that HA covering the sharp edges of a GO sheet significantly improved the compression resistance and lubricity of the composite, promoted cell proliferation, and improved biocompatibility. When immersed in a buffer solution, the quality of the composite hydrogels increased significantly, and spherical particles were deposited onto the surface, indicating that it effectively induced bone deposition and mineralization [17].

### 2.2. Nanocomposite Hydrogels from Polymer Nanoparticles

Polymeric nanomaterials include micelles, nanomaterials, core-shell particles, liposomes, and hyperbranched polymers. Among them, branched polymer nanoparticles have a large number of functional groups in the terminal region and, therefore, have higher reactivity, which can enhance the regularity and mechanical strength of the hydrogel network by crosslinking polymer chains via covalent or non-covalent bonds, a property which has attracted particular attention [18].

For example, Geiger et al. reported that [19] a PEGylated PAMAM dendrimer could improve improve delivery of growth factor for repair cartilage tissue. The addition of dendrimer at an appropriate concentration was conducive to the maintenance of the circular shape of encapsulated chondrocytes and the production of type II collagen and proteoglycans. In addition, in a later study, they used PEG as the core, selected different dendrimers containing carbamate and ester bonds as end groups, and synthesized several hydrogel materials for cartilage repair. The experimental results indicated that the compressive stiffness and viscoelasticity of carbamate crosslinked with dendrimer hydrogels were comparable to those of natural articular cartilage. Moreover, the material was injected into defects in the cartilage tissue as a macromolecular monomer solution and followed by light crosslinking and curing, which then produced a morphology more consistent with the defect [20]. The highlight of this method was that dendrimer nanoparticles containing different functional groups could be selected and refined by adjusting their concentration.

In addition, polymer nanoparticles can form a 3D hydrogel network above a certain concentration to provide support for local injectable and sustained cytokine release systems for joints. The use of thermosensitive polymer nanoparticles has a dual interaction with BMP-2. An injection and sustained bone morphogenetic protein 2 (BMP-2) release system achieved amphiphilic and hydrophobic interactions with BMP-2. A single injection of BMP-2 polymeric nanocomposite significantly promoted bone formation in the joints [21]. Nahideh et al. introduced growth factors loaded with gelatin to a matrix with β1-Polycaprolactone (PCL)-PEG copolymer nanoparticles. In vitro studies have reported that the scaffold had good biocompatibility and cell adhesion, and the expression of cartilage-specific ECM genes, such as type II collagen and aggrecan, results suggest that the scaffold effectively promotes the generation of ECM [22].

### 2.3. Nanocomposite Hydrogels from Magnetic Nanoparticles

Recently, magnetic response hydrogels have been widely used in biomedicine as intelligent hydrogels and to improve the biological activity of cells, tissues, and organs. Through the magnetic response to an external magnetic field, the corresponding functional structure was obtained remotely to regulate biochemical and mechanical properties [23,24,25,26,27] Recently, studies have shown that magnetic hydrogels could be used as a drug release and targeting system. For example, researchers [28] created a magnetic hydrogel based on ferric oxide in the ferromagnetic vortex domain and suggested that this unique magnetic hydrogel could significantly inhibit the recurrence of local breast tumors. Manjua et al. [29] developed a magnetic-response PVA hydrogel, which was driven by a magnetic field and non-invasive protein adsorption and movement, indicating that it had broad prospects for applications in tissue engineering, drug delivery, and biosensor systems. In addition, composite magnetic hydrogels prepared from self-repairing chitosan alginate gel and magnetic gelatin microspheres were used for tissue engineering and drug delivery [30]. Apart from magnetic hydrogels, various intelligent biomaterials activated by external stimuli such as light, pH, temperature, pressure, and charge have been developed. However, these non-magnetic, stimulus-responsive, smart biomaterials have long response times and less precisely controlled structures.

The main components of a magnetic hydrogel are the matrix hydrogel and the magnetic particles bound to the matrix. Iron-oxide-based magnetic nanoparticles (MNPs) with superparamagnetic properties and good biocompatibility have been incorporated into polymer matrices in the preparation of tissue-engineered magnetic-response hydrogels, such as γ-Fe_2_O_3_, Fe_3_O_4_, and cobalt ferrite nanoparticles (CoFe_2_O_4_) [31,32,33]. Magnetite (Fe_3_O_4_) is a compound with two iron sites, one-third Fe^2+^ and two-thirds Fe^3+^. The spacer charge transfer between Fe^2+^ and Fe^3+^ caused the absorption of the whole UV-Vis and infrared spectral region, resulting in a black appearance [34]. Maghemite (γ-Fe_2_O_3_) has a brownish orange color and is the oxidation product of magnetite (Fe_3_O_4_) at a temperature below 200 °C [35]. Previous studies have shown that a concentration of CoFe_2_O_4_ of 20% is toxic; however, at 10%, the toxicity is insignificant. In addition, due to a large number of nanoparticles, 10% (w/W) CoFe_2_O_4_ maximized the magnetic response in a preparation of biocompatible magnetic hydrogels [36,37]. Hermenegildo et al. [38] designed a new CoFe_2_O_4_/methacrylate gellan gum/poly (vinylidene fluoride) hydrogel, which created a promising microenvironment for tissue magnetic stimulation.

In addition, magnetic nanoparticles (MNPs), such as Fe_3_O_4_, can promote the proliferation and differentiation of MSCs. Nanohydrogels have been extensively studied as porous scaffolds for cartilage repair. For example, Chen et al. mixed superparamagnetic iron oxide particles with cellulose nanocrystals (CNC)/silk fibroin (SF) to prepare magnetic NC hydrogels. The degradation and cartilage regeneration of the material was characterized by multiparameter magnetic resonance imaging, which was a non-invasive method of detection [39]. Huang et al. used magnetic manipulation to mechanically stimulate MSCs to promote cartilage differentiation. The experiment was performed using gelatin β-magnetic NC hydrogels prepared from cyclodextrin and with Fe_3_O_4_ as the magnetic material. The results of the cell activity indicated that the magnetic hydrogel was biocompatible. The material and MSCs efficiently repaired cartilage under a pulsed electromagnetic field (PEMF) in rabbits. After 12 weeks, the regenerated tissue completely filled the defect and had the appearance of natural cartilage, according to histological staining [40].

At present, an advanced magnetic, smart hydrogel for cell embedding is commercially available from Micromod. Their biocompatible dextran-modified MNP can be integrated into a three-layer agarose scaffold. The magnetic hydrogel can be prepared by mixing and shows depth-dependent mechanics that enhance cell viability under magnetic stimulation and help maintain chondrocytes in a culture [41]. In addition, the multilayer tissues of 3D magnetic hydrogels with simulated structures, including magnetic particles coated with streptavidin (diameter between 10 and 12 nm), agarose, and type I collagen, can be constructed by 3D bioprinting. As compared to the traditional monolayer 3D matrix, 3D magnetic hydrogels have a similar structure to natural cartilage, and cells within them express more chondrocyte-related genes in vitro [42]. These new magnetic hydrogels highlight the potential application of cartilage replacement tissue at a specific location. Further studies are needed to assess the concentrations of MNPs and the matrix, the intensity and the type of magnetic fields, the MNPs modified by biological growth factors, and the ability of hydrogel composites to repair damaged cartilage in vivo.

Stem cell therapy has been used to treat cartilage injuries [43,44]. For this therapy to work, an important cytokine needs to be added to promote the differentiation of bone marrow mesenchymal stem cells into cartilage. Currently available cytokines, such as transforming growth factor-β3 and BMP2, have short half-lives [45] and less mineralization, respectively [46]. Kartogenin (KGN) is a small molecule compound that has been shown to induce chondrogenesis in bone marrow-derived mesenchymal stem cells (BMSCs) [47]. KGN was grafted onto the surface of MNPs, and the MNPs were then mixed with cellulose nanocrystals/dextran (CNC/Dex) hydrogels. In vitro and in vivo experiments showed that hydrogels loaded with KGN-MNPs enhanced the sustained release of KGN, raised host cells, induced cartilage differentiation of BMSCs, and effectively improved in situ cartilage regeneration [48].

Due to the high biocompatibility, strength, toughness, and cell adhesion of nano-HAP, many studies have reported that nano-HAP is effective for the repair of articular cartilage [49,50,51]. Magnetic nanocomposite hydrogels made from 1:0.5:10 nano-HAP particles, Fe_2_O_3_ nanoparticles, and PVA were prepared using ultrasonic dispersion and then freeze-dried by freeze–thaw crosslinking. Due to the Fe_2_O_3_ nanoparticles embedded in the hydrogel, the degradation experiments showed a relatively moderate-to-slow mass loss rate. As compared to isolated BMSCs, the activity of BMSCs in magnetic hydrogels did not change significantly, and the expression of chondrocyte-related genes was significantly increased [52].

### 2.4. Nanocomposite Hydrogels from Other Metal and Metal-Oxide Nanocomposites

Due to the unique physical properties of metal and metal-oxide nanoparticles, such as the conductivity of gold nanoparticles and the antibacterial properties of silver nanoparticles, nanoscaffold materials prepared using them have been widely applied in conductive scaffolds, switchable electronic devices, sensors, and driving devices [53]. In addition, they can also be used as imaging agents and drug delivery systems in biomedical applications.

Metal and metal-oxide NC hydrogels typically have excellent antibacterial, antiviral, and anti-inflammatory properties [54]. Their composite hydrogels have been commonly used in tissue engineering to treat wounds and to prepare wound dressings for wound healing. They also have important research value in cartilage tissue engineering. For example, Kumar reported that TiO_2_ nanoparticle-doped chitosan hydrogels improved density (1.287 g/cm^3^), loading capacity (13,475 N/m^2^), and fracture time, as compared with non-doped scaffolds. In addition, the addition of TiO_2_ nanoparticles slowed down the degradation of scaffold materials and facilitated the growth of seeded cells [55].

However, the biocompatibility of materials is the primary consideration for stent implantation. In this regard, Grant et al. compounded collagen with gold nanoparticles and implanted samples subcutaneously into pig ears for six months. Their results showed that the introduction of metal particles did not cause adverse tissue reactions, which indicated good biocompatibility, and that the service life of the material was longer than that of the control group (commercially available hydroxyapatite composite collagen) [56]. Although many studies have shown that precious metal nanoparticles such as gold and silver have effectively enhanced the physical and chemical properties of composite hydrogels, additional research to explore cellular behaviors in vivo is needed before further clinical applications can be attempted [57].

### 2.5. Nanocomposite Hydrogels from Inorganic Nanoparticles of Non-Metallic Origin

The introduction of larger inorganic nanoparticles into a flexible, organic polymer matrix forms an inorganic–organic network. The synergistic effect of the two has been shown to be conducive to improving the physical, chemical, and biological properties of NC hydrogels and has indicated their use in the preparation of functional or stimulus-responsive nanocomposite scaffold materials [58]. Inorganic nanomaterials commonly used in biomedicine include hydroxyapatite (HAP), silica, calcium phosphate, silicate β-Wollastonite, and other nanoparticles.

Among them, SiO_2_ nanoparticles have been of interest due to their inherent advantages, including a high modulus, large surface area, and diverse functions. For example, Xia et al. reported a composite hydrogel material using SiO_2_-grafted poly (butyl acrylate) (PBA) hybrid-latex particles and poly (acrylamide lauryl methacrylate) (P (Aam-co-LMA)) as the matrix. Due to the rigidity of the hard SiO_2_ core, it could bear more external force, and the hydrophobic chain segment of the soft PBA shell interacted with the hybrid-latex particles through physical aggregation and entanglement, which dissipated a significant amount of energy and enhanced the material’s mechanical properties (fracture stress, 1.48 Mpa; fracture strain, 2511%) [59]. The core hydrogel shell structure provided an effective way to improve the mechanical properties of scaffolds and indicated its potential in cartilage tissue repair.

In addition, some inorganic clays have been used to prepare nanoscaffolds. For example, Bonifacio et al. reported that the mechanical properties of gellan-gum hydrogel were improved by using mesoporous silica (MS) and sodium–calcium bentonite (BE). The results of the static compression tests showed that the Young’s modulus (BE-modified 112 + 9 kPa; MS-modified 140 + 25 kPa) was significantly higher than that of unmodified natural hydrogel material (86 + 5 kPa). In addition, in vivo experiments in mice showed that the introduction of MS did not produce a serious immune response and effectively enhanced the antibacterial properties of the scaffold materials [60].

### 2.6. Exosome-Loaded Hydrogel

Exosomes are endogenous nanovesicles with a diameter of approximately 30–150 nm. They have biological functions similar to those of maternal cells. They often act as a medium for cell-to-cell communication and play an important role in both physiological and pathological processes. Studies have shown that stem-cell exosomes can promote tissue repair as well as wound healing and have a significant potential in cartilage tissue repair [61,62,63]. In particular, exosomes can be modified for targeted drug delivery [64,65,66,67,68].

Hydrogels loaded with stem-cell-derived exosomes for cartilage repair have a significant potential in clinical applications [69]. It also promoted the effects of both exosomes and hydrogels on cartilage defects, to achieve synergistic treatment. Liu et al. prepared a light-induced crosslinked hydrogel loaded with exosomes, which was crosslinked in situ and integrated with the defect post-injection, and it had good biocompatibility. The repair ability of the hydrogel loaded with exosomes was evaluated using a rabbit model exhibiting knee-joint defects. The research results showed that the network structure of the hydrogel effectively encapsulated the exosomes and could permanently exert positive regulation on the surrounding and migrating cells. The cells were integrated with the surrounding natural cartilage layer; the results of immunohistochemical staining show that the newly formed tissue was almost hyaline cartilage, which has the same composition as the body’s own cartilage [70]. In addition, the development of a new co-delivery system based on TGF-β1-loaded RGD coupled with alginate microspheres has been reported. The hydrogel carrier was loaded with microencapsulated odontogenic mesenchymal stem cells at a density of 2 × 10^6^ cells/mL of alginate solution. Histochemistry and immunofluorescence staining confirmed that the hydrogel scaffolds transplanted with exosomes promoted cartilage regeneration, which indicated its potential use in the reconstruction of temporomandibular joint discs and the application of appendage bones [71].

However, there are still many issues to be resolved for the use of exosomal nanohydrogel scaffolds in clinical applications, including how to obtain large amounts of exosomes and improve their antibacterial ability and repair effects, how to maximize the use of stem-cell exosomes to promote cartilage repair, and how to use the cavity structure of exosomes to load appropriate drugs, etc. In-depth experimentation concerning the development of exosomes, the selection of hydrogel materials, and the mechanisms of cartilage repair will be needed before hydrogels loaded with stem-cell exosomes can be used in the clinical treatment of cartilage trauma. 

## 3. Crosslinking of Nanocomposite Hydrogels

The structure and the function of nanohydrogels can be designed and adjusted by selecting nanohydrogels with different physical, chemical, and biological properties. The polymer-chain structure of a nanohydrogel contains many active functional groups that can be physically and chemically crosslinked. Nanomaterials are biodegradable and have controllable mechanical properties and characteristics [72]. Hypoxia is a key feature in the microenvironment of RA, which is caused by inflammation, the obstruction of oxygen delivery, and microvascular repair. In order to reduce oxidative stress in the inflammatory microenvironment, many antioxidants appear in the inflammatory region, including glutathione (GSH). Therefore, we could use a similar strategy to create stimulus-responsive nanogel carriers for RA treatment. Peptide-reductone nanogels linked to mPEG-NH2 could allow for the intelligent release of methotrexate (MTX) at the inflammatory site in RA.

Seo et al. [21] used thermosensitive polymer nanomaterials with dual-interaction forces to prepare injectable hydrogels that continuously released BMP-2. In this study, thermosensitive polyphosphonitrile was the backbone material of the hydrogels. Hydrophobic isoleucine ethyl ester and hydrophilic polyethylene glycol were introduced into polyphosphazene to form double-interacting polymer nanoparticles (D-NP). BMP-2 was difficult to absorb, but when it reacted with D-NP’s dual-interaction surface, BMP-2-D-NPs were eventually assembled with hydrophobic parts and ionic interaction groups. They could not only perform BMP activities in vitro, but they also formed in situ nanocomposite hydrogels in vivo. Injected BMP-2-D-NPs nanocomposites at the top of the cranial bone sensed body temperature to form hydrogel scaffolds in situ. This novel strategy allowed for sustainable BMP-2 release and produced a new bone layer in vivo. Liu et al. fabricated injectable nanocomposite hydrogels by a photo-crosslink. These hydrogels were synthesized by incorporating methacrylic-anhydride-modified poly(amidoamine) (PAMAM) dendrimer as a nanomaterial that changed the physicochemical properties and mechanics of the hydrogel performance. It also enhanced the chondrogenic differentiation of rabbit adipose derived stem cells (rASCs) in vitro. Moreover, this hybrid nanohydrogel successfully promoted cartilage regeneration in vivo [73].

Natural tissues and organs have features at a nanoscale where cells directly interact with nanostructured ECMs. Therefore, the biological properties of nanomaterials are important in cell growth and tissue regeneration [74]. Nanoscale and microscale scaffold structures are also essential for cartilage tissue engineering. Nanofibers and nanomaterials with nanostructured surfaces have been used to simulate the ECMs of cartilage [75]. Cartilage tissue contains not only chondrocytes, but also dense nanostructured ECMs rich in proteoglycans, collagen, and elastin fibers. Therefore, nanostructure characteristics have endowed nanohydrogels with useful biological properties [74].

However, hydrogels require significant hydration and have poor mechanical properties, which have limited their use in vitro and in vivo. To overcome these shortcomings, they have been combined with different materials [76]. For example, nanomaterials have been inserted into the matrices of hydrogels to broaden their application, so current research on hydrogels has focused on the development of nanomaterials [77]. Nanoparticles containing organic and inorganic substances, such as HAP, clay, graphene, and metal nanoparticles, have been used as filling materials to enhance the mechanical strength [78] of hydrogel matrices. Nanoparticles have a larger surface area–volume ratio, which increases surface reactivity, the release of bioactive agents, and bioavailability, as well as improves their mechanical properties. In addition, since they can penetrate tissues through capillaries and epithelial cells, they have improved transport characteristics and effectively delivered therapeutic agents to target cells [79,80,81]. As compared with the independent use of nanoparticles, nanoparticles formed with hydrogels have had advantages. For example, when particles were embedded in a polymer matrix during scaffold crosslinking, they had a more uniform distribution, and these nanoparticles also showed improved drug stability and high efficacy when the drug was activated.

## 4. Fabrication of Nanocomposite Hydrogels

**Figure 2:**
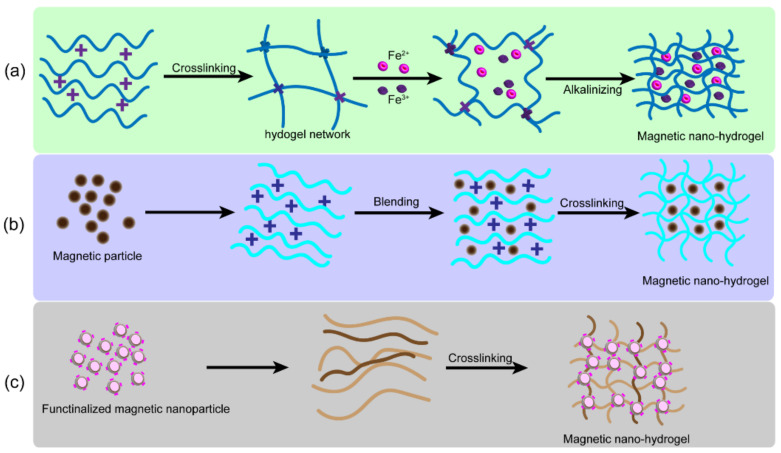
Schematic diagram of three main synthetic methods for preparing magnetic hydrogels. (**a**) In situ precipitation method. (**b**) Blending. (**c**) Grafting.

Since the interaction between nanoparticles and hydrogels has played an important role in the properties of composite hydrogels, the dispersion of nanoparticles in hydrogels and their crosslinking with polymer chains have been key considerations in the preparation of nanoscaffolds [82]. Table 1 shows the representative fabrication methods to prepare nanocomposite hydrogels. Figure 2 illustrates three main fibrication methods for preparing magnetic hydrogels. Praveen et al. [83] divided nanohydrogels into five categories according to their preparation processes. In method 1, the monomer, crosslinking agent, and nanoparticles were mixed in suspension to gelate into nanohydrogels. In method 2, after gelation, the nanoparticles were physically embedded in the hydrogel matrix. In method 3, the nanoparticle precursor was used to prepare nanoparticles as pre-gels. In method 4, crosslinked nanoparticles were used to form hydrogels. Finally, in method 5, the hydrogels were prepared by adding different gel molecules into nanoparticles and polymers. The applications of the nanomaterial determined the preparation method. 

**Figure 3:**
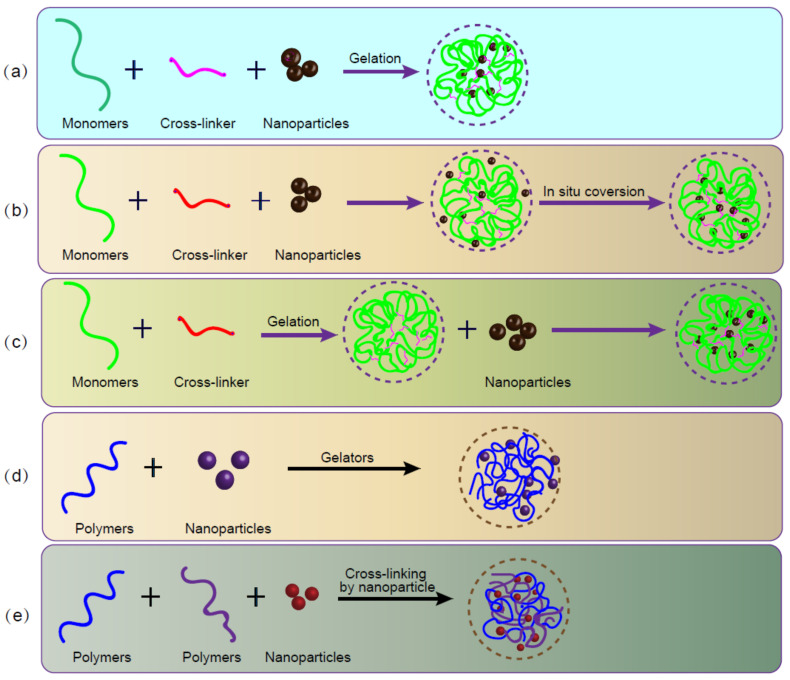
Schematic showing five main methods for the preparation of nanoparticle-hydrogel composites: (**a**) formation of hydrogels in nanoparticle suspensions; (**b**) formation of reactive nanoparticles within precast gels; (**c**) formation of nanoparticles physically embedded in the hydrogel matrix; (**d**) crosslinking using nanoparticles to form hydrogels; and (**e**) nanoparticles, polymers, and physical gelling agents to form hydrogels.

Figure 3 illustrates five conjugate approaches to obtain nanoparticle hydrogels. At present, the most widely studied methods of forming nanohydrogel scaffolds have included injectable nanohydrogel molding, 3D printing nanohydrogel molding, and electrospinning nanocomposite hydrogel molding. The first two are usually formed from pre-gelatin and can be classified as methods 1, 3, 4, and 5, after crosslinking. 

### 4.1. Injectable Nanohydrogel

**Figure 4:**
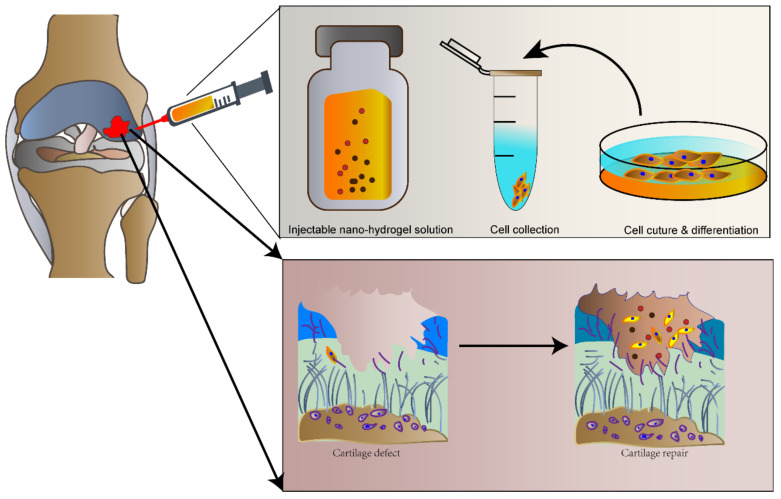
Injectable hydrogels for cartilage repair. The injectable nanohydrogel complex promotes regenerated cartilage tissue to repair cartilage defects or worn tissues in vivo.

Injectable nanohydrogels have been used for minimally invasive in situ repairs of cartilage defects. Seed cells were directly injected into the defect site with a growth-factor-hydrogel scaffold material (Figure 4). As compared with pre-formed scaffold materials, injectable hydrogels have been more advantageous for osteochondral tissue engineering as they have been easier to apply in clinical settings and patient compliance has been higher. They are also less invasive and have a strong targeting ability. They can be directly delivered to large irregular or geometrically complex defect sites for repair. Since they are patient-friendly, they have been widely used for cartilage tissue repair [101]. There have been many reports on the application of injectable hydrogels in osteochondral tissue engineering [102,103].

In recent years, many studies have been conducted concerning the preparation of NC hydrogels through injectable hydrogels to form scaffold materials. For example, Lee et al. used a polyethylene glycol poly L-alanine polyaspartic acid (PEG-PA-PD) three-block copolymer as the matrix and prepared injectable hydrogel scaffolds by introducing the chondrocyte-inducer kartogenin and layering double-hydroxide (LDH) nanocomposites with refined glyceryl aspartic acid. Tonsil-derived mesenchymal stem cells (TMSCs) were used for in vitro experiments. As compared with a pure water gel system, the addition of the nanocomposite system improved cell aggregation and significantly enhanced the expression of cartilage biomarkers. In addition, various other bioactive molecules have been used to modify the surfaces of LDHs by ion interaction to design and prepare injectable NC hydrogels [104] that have been used for cartilage repair.

However, for the clinical application of injectable nanohydrogels, we must improve its mechanical properties. Kuang et al. reported the preparation of an injectable NC hydrogel by introducing in situ calcium phosphate nanoparticles and poly-L-glutamic acid (PGA) into the methylamino ethyl methacrylate (DMAEMA) and 2-hydroxyethyl methacrylate (HEMA) matrix. The carboxyl groups of PGA interacted with the tertiary amines of the DMAEMA fragments, thus enhancing the mechanical strength of the hydrogels. As compared to the in situ prepared hydrogel, the NC hydrogel had a suitable injection time, and the material’s tensile strength and fracture energy increased by 321.1 kPa and 29 kJ/m^2^, respectively. It also significantly promoted cell adhesion and differentiation. In vivo experiments showed that the NC hydrogel had a better osteogenic effect than the pure matrix hydrogel without nanoparticles, and the methacrylate group on its chain was easily functionalized by aptamers. Both in vivo and in vitro experiments promoted the identification and capture of bone marrow stromal cells and facilitated regeneration [105].

### 4.2. 3D Bioprinting Nanohydrogel

Natural cartilage tissue has layers. In order to generate artificial materials with similar mechanical properties, it has been necessary to study and simulate the internal layered structure of cartilage. The joint soft gradient has been extensively studied. Double-layer and multilayer hydrogels with a gradient hardness can better repair cartilage and subchondral bone defects. In addition, 3D bioprinting technology has been effective for preparing layered materials. Common 3D bioprinting technologies used in bone-tissue engineering have included stereolithography appearance (SLA), selective laser sintering (SLS), fused deposition modeling (FDM), ink jet printing, and bioink [106,107].

Most recent studies have used 3D bioprinting technology to prepare NC hydrogel scaffolds that have been used for cartilage repairs [108]. For example, Park et al. used glycidyl methacrylate (GMA)-modified silk fibroin (Sil-MA) hydrogel as a biological ink and showed show that Sil-MA hydrogels were biocompatible, and their complex structures could be prepared by 3D bioprinting [109,110]. Zhou et al. prepared GelMA and hyaluronic acid methyl acrylate (HAMA)-composite hydrogel scaffolds by means of photo-crosslinking 3D bioprinting technology. The stent had a fine, controllable macroscopic shape and an internal pore structure. The experimental results showed that chondrocytes could bind to the scaffold and regenerate mature cartilage with cartilage-specific ECMs [111,112].

The introduction of nanoparticles not only has improved the mechanical properties of scaffold materials, but it has also promoted cartilage repair by controlling the release of growth factors. For example, Castro et al. introduced hydrothermal-treated n-HA and TGFβ1 immobilized PLGA nanospheres with a core shell structure in a PEG matrix. Two nanohydrogels were prepared via 3D bioprinting. As compared to commercial printers, the desktop light-curing printer was inexpensive, with high precision and a multi-layer complex structure. The size of HAP nanoparticles was 80–100 nm, and the diameter of PLGA nanospheres was 75 + 17 nm. The experimental results showed that the NC hydrogel containing HAP was more conducive to hMSC proliferation than the pure hydrogel of the control group, and its compression modulus increased by 29%, which was indicative of improved mechanical strength. The nanohydrogel containing transforming growth factor beta 1 (TGFβ1), incorporated into microparticles of blends of poly Lactic-co-Glycolic Acid (PLGA) nanospheres, contributed to the long-term recovery and growth of cartilage tissue [113].

The structural design of scaffolds has played an important role in 3D bioprinting. For example, Meng et al. used PVA as a matrix, introduced GO and HAP nanoparticles, and prepared cartilage repair scaffolds with a gradient of hardness via extrusion 3D bioprinting. The designed support material had a unique structure: the surface was smooth and flat without print-line spacing, which was conducive to load-bearing. The distance between the scaffold and the lower line increased layer by layer, which was conducive to the formation of a firm connection between the material and the bone base. At the same time, there was no interface between the layers, which prevented the sample from loosening and falling off after implantation. The porosity of the material was also graded due to the temperature gradient of the manufacturing process: the temperature from substrate to surface was increased layer by layer, which slowed down the growth of nucleation and decreased the pore gradient of the hydrogel. In addition, the study suggested that the addition of GO-HAP nanoparticles reduced the intermolecular hydrogen bonding and the molecular entanglement density, which enhanced the dynamic viscosity of the hydrogel. The solution showed obvious shear thinning within the range of the printing shear rate, which effectively avoided extrusion expansion and thus improved the printing application range and accuracy of the samples [99]. Recent studies have synthesized a nanoscaffold for MSC exosome delivery by 3D printing chondrogenic extracellular matrix (ECM)/gelatin methacrylate (GelMA)/exosome using desktop 3D printing. These nanoscaffold materials were implanted in a rabbit model with an osteochondral defect, and the results suggested it promoted cartilage regeneration and repaired the osteochondral defect [87].

The condition parameters of 3D bioprinting also influences the materials that can be used. For example, Gao et al. used coaxial-nozzle-assisted 3D bioprinting to manufacture a subchondral scaffold and prepared double-layer scaffolds by extrusion molding, low-temperature deposition, 3D bioprinting, and UV curing. In this report, rat mesenchymal stem cells (rMSCs) and rabbit bone cartilage were used for in vitro and in vivo experiments, in which they measured the effects of line spacing and the width of 3D bioprinting fibers on the pore size, the porosity, the specific surface area, the mechanical strength, and the cartilage repair ability of the scaffolds. The results showed that as the line spacing increased, the mechanical properties of the material gradually decreased, while the cartilage repair ability first increased and then decreased. Therefore, it is important to select appropriate printing parameters in 3D bioprinting [114].

In addition, cartilage repair scaffolds have been prepared using 3D bioprinting of a polymer melt. For example, Shalom et al. reported that WS2 inorganic nanotubes (ws2-nt) were dispersed in PLA by melt extrusion for fuse-manufacturing (FFF) 3D printing. The results showed that ws2-nt improved the mechanical properties of printed PLA, such as the elastic modulus, the yield strength, and the fracture strain, which were increased by 20%, 23%, and 35%, respectively. In addition, after the FFF treatment, the dispersion of ws2-nt in the matrix was better than that in the preprinted filaments. As compared to the polymer solutions, the molten polymer solidified slowly and had higher crystallinity, so it had a higher modulus. The material was expected to be further used in cartilage tissue repair [115]. As compared to hydrogel scaffolds with polymer aqueous solutions, the mechanical properties of polymer scaffolds have been better, but their biocompatibility has often been poor. Their application in cartilage tissue repair remains challenging.

### 4.3. Electrospinning of NC Hydrogels

In natural tissues, the extracellular matrix plays an important role in regulating cell behavior and function. Therefore, scaffold materials similar to the extracellular matrix in structure can provide a microenvironment suitable for repair and the regeneration of damaged tissues. The extracellular matrix of cartilage is mainly composed of a type II collagen network and feather-like glycoproteins, which provide sufficient mechanical strength for cartilage tissue and nutrition, as well as the transport of metabolites for chondrocytes [116]. The structure of nanofibers is similar to that of a collagen-fiber network. They not only provide appropriate mechanical properties, but they also provide a microenvironment for cell adhesion, migration, proliferation, and differentiation. Studies have shown that cells are round or oval [117] in nanofibers and hydrogel scaffolds. This form of chondrocytes is more similar to that found in a natural cartilage matrix, which is more conducive to maintaining the normal phenotype of chondrocytes. Round or oval stem cells have supported the differentiation into chondrocytes [117]. In addition, nanofibers have a high specific surface area, which has been conducive to the release of bioactive substances, such as proteins, drugs, nucleic acids, etc., transmitted through fibers [118].

Electrospinning consists of spin-spraying a polymer solution or melt through the action of an electric field to prepare one-dimensional nanofibers. It has attracted interest in the fields of tissue engineering, drug delivery, medical diagnosis, environmental protection, protective clothing, and dental materials. Common electrospinning equipment consists of three parts [119], namely, a high-voltage power supply, a metal needle syringe, and a grounding device. Nanofibers prepared by electrospinning have been widely used in cartilage tissue engineering due to their relatively uniform diameter and adjustable structure. For example, Scaffaro et al. prepared PCL composite nanofiber scaffolds with different concentrations of GO and surface-grafted PEG modifications (go-g-peg) by electrospinning that had a high mechanical strength and good cell compatibility, as well as being promising for use in cartilage tissue repair [120]. Chen et al. used coaxial electrospinning technology to coat glucosamine sulfate (GAS) obtained by chitin hydrolysis with PCL to prepare PCL/GAS composite nanofibers. The sustained release of GAS in the scaffold was conducive to the growth and proliferation of chondrocytes and was expected to be further used for cartilage tissue repair [121]. Silva et al. prepared and characterized coaxial polyglyceride sebacate (PGs)/PCL electrospinning scaffolds to simulate the nanostructure and arrangement of collagen fibers in articular cartilage ECMs. Compared with uniaxially oriented PCL scaffolds, coaxial PGs/PCL-aligned nanofibers promoted the sustained release of the osteocyte-inducer KGN [122].

Highly oriented nanofiber and hydrogel systems can be combined to form complex cell-compatible hydrogel fibers as composite scaffold materials for use in tissue engineering. When the technology has been applied to the preparation of nanohydrogels, it combined the advantages of electrospun fibers and hydrogels to provide a more competitive approach [14] for the preparation of scaffolds. When using nanocomposite hydrogels for tissue engineering that have been formed by electrospinning, we need to consider the microstructure of polymer materials, the composite fiber network, the permeability of the scaffold, the toxicity of the material, and the adhesion of cells, among other factors. The microstructures of electrospun fibers and nanoparticles are usually the following: single component nanoparticles or an electrospun fiber layer, the molecular scale composite nanoparticles and the electrospun fiber layer, the coaxial electrospinning composite fiber net, the particle-reinforced hydrogel, and the particle-reinforced electrospun fiber layer. The composite structure of electrospun fiber and hydrogel scaffolds have been divided into several types: the block structure of hydrogel-coated fiber, hydrogel and fiber stacking structure, bulk-electrospun fiber structure with gel deposition, and tubular-wound fiber and hydrogel structure [123].

For example, Hejazi et al. prepared PCL/gel/n-HAP for bone regeneration and CS/PVA soft five-layer-gradient nanofiber scaffolds for cartilage regeneration by electrospinning [97]. Rajzer et al. prepared PLA fiber-layered stacking scaffolds by electrospinning and 3D printing technology, into which gelatin hydrogel was injected. The experimental results showed that the scaffold could be used for the reconstruction of nasal cartilage and subchondral bone [124]. Bas et al. used highly negatively charged star PEG/heparin hydrogel (PEG/Hep) as a substrate and prepared NC hydrogels by combining them with medical PCL melt-electrospinning fiber networks. The material showed anisotropic, nonlinear, viscoelastic, and other mechanical properties similar to natural material, and it enabled human chondrocyte cultures and new cartilage formation in vitro. In addition, the material was simulated by the p-type finite element method to study the overall balance and dynamic, instantaneous biomechanical properties of cartilage tissue [125]. Guo et al. reported that SF and PCL were compounded by electrospinning and prepared Sr^2+^ hydrogel meniscus scaffolds using Sr^2+^ as an active factor. The experimental results showed that the scaffold effectively protected cartilage and delayed the development of osteoarthritis without introducing seed cells [126].

Although synthetic materials containing nanofibers with controllable structure are easy to prepare by electrospinning, these materials lack bioactive sites necessary to interact with cells. Therefore, nanofibers prepared from natural polysaccharides and proteins for tissue-engineered cartilage have become a research hotspot. In recent years, polypeptide self-assembled nanofibers have also been used for cartilage repair due to their composition and structure being closer to that of the natural cartilage extracellular matrix [127] J. Kisiday et al. found that the self-assembled nanofibers from KLD-12 could be combined with chondrocytes. Thus, chondrocytes can maintaine their morphology in the hydrogel and secreted cartilage extracellular matrix components’ mucopolysaccharide and type II collagen [127].

There have also been related studies on the combination of natural biomaterials and electrospinning to prepare cartilage-repair scaffolds. For example, Chen et al. used an acellular matrix (CDM) of bovine scapular cartilage as the matrix and prepared the gelatin/polylactic acid short fibers by electrospinning for use as reinforcement materials and then prepared scaffold materials for cartilage repair by 3D printing [128]. Feng et al. successfully prepared cartilage-derived extracellular matrix (CEM)/PCL composite nanofiber scaffolds by electrospinning. CEM was prepared by grinding cartilage slices into powder and digesting them before filtering them into a looser structure. The scaffold was biocompatible and non-cytotoxic, and it effectively promoted chondrocyte proliferation [129].

## 5. Conclusions and Prospects

There are still many challenges in the development of cartilage tissue engineering, and biomimetic nanoscaffold materials are an important development in cartilage-tissue-engineering scaffold materials. The similar molecular structure to cartilage ECMs can maintains the growth and stability of the chondrocytes. In the process of biomaterial research and development, biological or chemically-modified composite nanomaterials, injectable materials, and biomimetic materials will be an important part of future research.

Most of the scaffolds used in articular cartilage are made of natural materials and have been studied in many clinical trials, and the mean clinical scores of the clinical literature for scaffold technology have been significantly improved, as compared to the preoperative values. More than 80% of patients have an improved prognosis. However, there has not been a consensus on the best cartilage repair material. Randomized clinical trials and longer follow-up periods are needed to obtain information on the clinical effectiveness of scaffold-based tissue-engineered cartilage repair. Although the application of nanocomposite scaffolds/hydrogels has been routine in animal practices, it has not been performed in clinical trials.

With the development of nanotechnology and the in-depth research on the microenvironment of cartilage, the biocompatibility, the simple preparation, the high drug-loading efficiency, the multiple stimulus responses, the controllable drug release, and the mechanical properties that promote nanocomposite hydrogels have been widely used in cartilage tissue repair.

Since the cartilage extracellular matrix has a highly ordered, multiscale structure, ranging from supramolecular to nanoscale, nanoparticles with this multiscale structure and the corresponding biological functions of each scale could become an ideal material for cartilage repair. Nanoparticles controlled by injection molding, 3D printing, electrospinning, and other methods have been effectively dispersed in nanohydrogel, and the surface of the nanohydrogel has also been modified in various ways to build a multifunctional cell-growth-factor delivery platform. To realize the combined therapy of multiple growth factors or imaging-guided multiple therapies, nanocomposite hydrogels can serve as drug delivery materials for the repair of cartilage tissues. By modifying the surface of multifunctional molecules, multifunctional drug delivery can be developed to maximize the loading and the release of drugs for treating joints while simultaneously extending the retention time. These unique characteristics of nanohydrogels indicate their potential in controllable drug-release delivery systems.

Therefore, nanomaterials and hydrogels have unlimited possibilities and will be further explored in future research. Through template molding, self-assembly, microfluidics, and 3D-printing technologies, building ultrastructures or dispersing nanoparticles in hydrogels to form composite materials can significantly improve the performance of hydrogels.

## Figures and Tables

**Table 1:** Recent examples of hydrogels with nanomaterials for articular cartilage repair.

Nanoparticles	Hydrogel	Preparation Method	In Vitro/Vivo Model	Function or Potential Applications	Ref
Carbon nanotube	Molten agarose	Two-dimensional culture	Three-dimensional (3D) pellet cultures	Promote chondrogenic ECM	[84]
Micelle	Methacrylated hyaluronic acid (MeHA) hydrogels	Photo-crosslink	Rabbit cartilage defects	Improve the mechanical properties: low swelling, effective self-recovery, and efficient energy dissipation	[85]
Polymersome	β-CD-grafted hyaluronic acid macromer	Crosslinking	Rat model of knee osteoarthritis	Localized and sustained drug release	[86]
Exosomes	Porcine cartilage/GelMA	3D desktop-stereolithography technology	Rabbit model of osteochondral defect	Promote chondrocyte migration and cartilage regeneration	[87]
Human umbilical cord mesenchymal stem cell-derived small extracellular vesicles (hUC-MSCs-sEVs)	Gelatin methacrylate (Gelma)/nanoclay hydrogel (Gel-nano)	Chemical crosslinking by ultraviolet radiation	Full thickness cylindrical cartilage defects in rats	Promote cartilage regeneration	[88]
Titanium oxide (TiO2), carbon nanotubes (CNTs)	polyacrylamide (PAM)	Free radical polymerization reaction	In vitro evaluation	Enhance mechanical behavior and puncture resistance	[89]
Chitosan (CS) nanoparticles	Silk fibroin (SF) hydrogel	Ultrasound-induced crosslinking	Rabbit model of knee cartilage defects	Stimulate chondrogenic of BMSC and repair articular cartilage defects	[90]
Sr-doped hydroxyapatite (HAP) microspheres	RGD-alginate	Crosslinking	In vitro model	Bone repair	[91,92]
Nano-hydroxyapatite/poly(vinyl alcohol) hydrogels	Poly(lactic-co-glycolic acid)/nano-hydroxyapatite/poly(vinyl alcohol)	Crosslinking by freeze–thaw	In vitro culture	Promote chondrogenesis	
Magnetic nanoparticles	poly(vinyl alcohol) and nano-hydroxyapatite (n-HAP),	Ultrasonic dispersion method and freeze–thaw crosslinking	In vitro model	Promote proliferation and differentiation of the BMSCs	[52,93]
Magnetic nanoparticles	PLGA/Col-I-PLGA/n-HAP	Low-temperature deposition manufacturing	In vitro model	Cell compatibility	[94]
Zinc oxide	Polycaprolactone (PCL)	Electrospinning technique	In vitro model	Enhance osteochondral differentiation	[95]
Poly(amidoamine) (PAMAM) dendrimers	gelatin methacrylate (GelMA) hydrogel	Photo-crosslink	Rat knee cartilage defect	Promote cartilage tissue regeneration	[73]
Biodendrimer	PEG3400-(PGLSA-MA4)2 macromer	UV–photo-crosslink	In vitro model	Promote cartilage regeneration	[96]
n-HAP	PCL/gelatin	3D printing	In vitro cytotoxicity evaluation	Promote MSC proliferation	[97]
Graphene oxide (GO)	Gelatin hydrogel	Microplasma-assisted crosslinking method	Rat model of cartilage defects	Promote formation of healthy hyaline cartilage	[98]
GO	PVA/HAP	3D printing	In vitro model	Artificial cartilage replacement	[99,100]

## Data Availability

Not applicable.
